# Monopole antenna array design for 3 T and 7 T magnetic resonance imaging

**DOI:** 10.1371/journal.pone.0214637

**Published:** 2019-04-01

**Authors:** A. S. M. Zahid Kausar, David C. Reutens, Ewald Weber, Viktor Vegh

**Affiliations:** 1 Centre for Advanced Imaging, University of Queensland, St Lucia, Brisbane, Australia; 2 School of Information Technology and Electrical Engineering, University of Queensland, St Lucia, Brisbane, Australia; University of California San Francisco, UNITED STATES

## Abstract

**Purpose:**

Ultra-high field magnetic resonance imaging poses a number of challenges for robust radio frequency coil designs. A monopole antenna array can potentially overcome key limitations of birdcage coil designs and may provide a useful radio frequency coil for brain imaging.

**Methods:**

Four, 8 and 12 element monopole antenna arrays were simulated using 3 T and 7T magnetic resonance imaging frequencies. For comparison, 4, 8 and 12 element birdcage coils were also simulated. Coil performance was evaluated and compared and the impact of shielding was assessed. A 4 element monopole antenna array was fabricated and bench tested.

**Results:**

Comparison of the 4, 8 and 12 element designs suggest that the monopole antenna array leads to better field properties than the birdcage coil in all configurations studied: unloaded, loaded with saline and loaded using a head phantom. Improvements in field properties and homogeneity were evident at both field strengths, implying that the monopole antenna array has potential for head imaging. The monopole antenna array also appears to be more efficient than the comparable birdcage coil design. Additionally, the former is scalable via the addition of more elements whereas our results suggest that this is not the case for the latter. Bench testing results show that the monopole antenna array is well matched with the transmission line, and mutual coupling between elements is sufficiently low.

**Conclusion:**

We found the monopole antenna array generated a larger field intensity than the birdcage coil design, whilst also producing a more useful magnetic resonance imaging field as measured by radio frequency field homogeneity. Our study suggests that magnetic resonance imaging of the brain can likely benefit from the use of radio frequency monopole antenna arrays.

## Introduction

Radio frequency (RF) coils are an essential component of a magnetic resonance imaging (MRI) system. They are used for pulse transmission and signal detection, and can be tailored to optimise imaging of specific body parts [1, 2]. Magnetic resonance image quality is affected by the function and design of the RF coil, hence considerable effort is expended on developing coils for specific applications. RF coils must be customised to specific frequencies and scanners as RF coils are made to transmit and receive time varying magnetic fields. These fields vary around the scanner Larmor frequency, as defined by the scanner field strength. To achieve a certain frequency, given limitations on physical size, and to improve coil efficiency and allow for tuning and matching, capacitive and inductive components are routinely used in RF coil designs [3–6]. As a consequence, coil layout can differ vastly between different designs and applications. Prominent designs include surface coils with a limited field-of-view but high sensitivity, volume coils such as the birdcage coil (BCC) with a comparatively large field-of-view but with decreased sensitivity, and transverse electromagnetic coils which are tailored to ultra-high field applications.

Transmit and receive body coils currently used in cylindrical MRI scanners are BCCs. The BCC comprises of two circular conductive loops or end rings, connected by an even number of equally spaced conductive elements referred to as rungs, legs or elements. The number of rungs generally changes with coil size and frequency. Capacitors are used in the end ring and often in the rungs as well, to be able to set the coil size and frequency. In transmit mode a sinusoidal current is applied at the end rings and a homogeneous magnetic field is formed around the Larmor frequency in the field-of-view of the coil [7].

BCC construction becomes more challenging as operating frequency increases with increasing MRI scanner field strength [8], because the product of coil capacitance and inductance must be reduced. At high frequencies (3 T MRI and above) individual coil rungs become increasingly sensitive to changes in loading and impedance. Changes in rung impedance may result in deterioration of the transmit RF field or reduction in sensitivity. This problem can be overcome by reducing the coil size and incorporating tuneable capacitors into the rungs. However, this solution limits the practical use of the coil as either the coil field-of-view becomes too small or tuning and matching is required on each rung for each scan [8, 9]. The use of variable capacitors also leads to increased parasitic inductance and hence degrades the efficiency of the coil.

The physical size of the BCC is inversely proportional to the signal-to-noise ratio achieved in the field-of-view [10]. For brain imaging, tailoring a BCC to the human head, provides significant benefits in terms of MRI quality. However, the relatively large number of capacitors in BCC designs makes them vulnerable to RF interference, particularly at high field. Shielding can help to reduce RF interference but also decreases the signal-to-noise ratio. Recently, a one-sided dipole antenna [11] and an array of dipole antennae [12] were described for 7 T MRI. The 50 cm dipole antennae were too long for human brain imaging applications as the shoulders prevented access to the centre of the field-of-view. A folded design was introduced to overcome this limitation [11] and, the substrate between the antennae and skin was later manipulated to reduce reflected waves [12]. Electromagnetic radiation losses were shown to occur near the folds. Ultimately, this design is impractical as the substrate has to be in direct contact with the skin to be effective. Whilst monopole arrays have been characterised for a wide range of applications including MRI [13–17], their use in MRI has not been extensively investigated. Features such as a simple structure, low cost, ultra-wideband characteristics and omnidirectional radiation patterns make them attractive for MRI applications. An extended monopole antenna array (MAA) for 7 T MRI with individual shields was described recently [18]. Capacitors in each of the monopoles were used to increase the field-of-view and improve field uniformity in the axial direction. Although this design shows promise, the relatively large mutual coupling between monopoles impacts upon coil efficiency. An initial investigation proposed the use of a simple monopole array for 7T MRI and performance was measured against a loop coil [19]. However, the monopole array could only be placed down to the shoulders, limiting its use for full brain imaging. In addition, eddy currents were also identified to be excessive based on the design.

Our aim is to further investigate the utility of a monopole array for head imaging at 3 T and 7 T MRI. We simulated 4, 8, and 12 element monopole array designs at both 3 T and 7 T MRI frequencies. We benchmarked MAA performance against BCC simulations and made comparisons with the results previously published for the dipole antenna array design. Benching testing was performed on a fabricated 4 element MAA.

## Methodology

### Theoretical considerations

**Fig 1:**
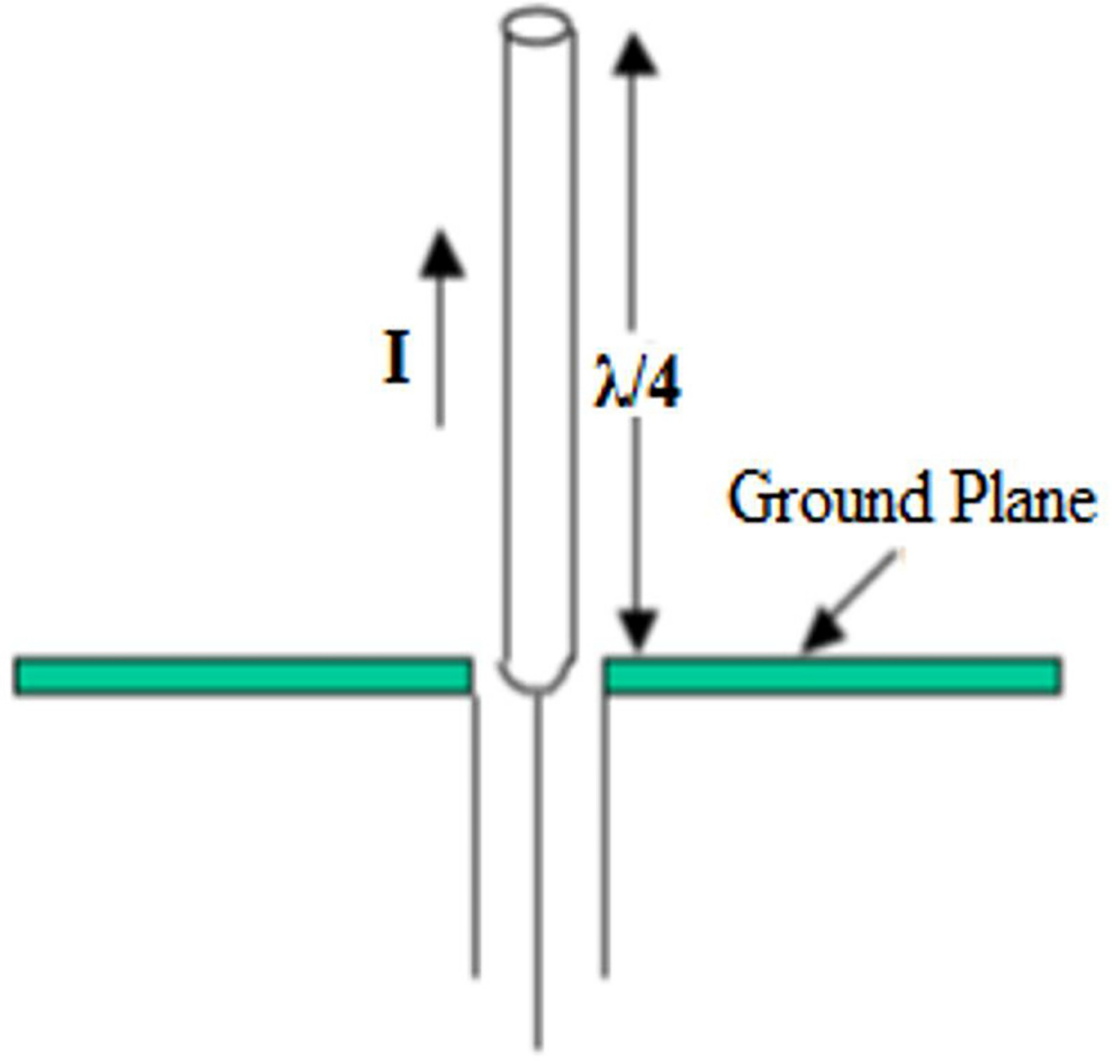
The geometrical specifics of quarter wave monopole antenna.

Monopole antennae are the simplest kind of RF antennae and comprise a single linear element, with the length of the monopole being equal to the quarter wavelength (Fig 1). The length (L) for of the monopole antenna is defined as [19]:

$$L_{\mathrm{monopole}}=\frac{1}{4}k\lambda_{0}=\frac{1}{4}k\frac{c}{f},$$ (Eq 1)

where *λ*_0_ is the wavelength, *c* is the speed of light, *f* is the frequency and *k* is used to adjust the length of the monopole. Notably, *k* compensates for propagation speed and is close to 1 if the wire diameter is thin compared to the free-space wavelength [20–23]. A MAA can be created using a number of individual monopoles.

In contrast, the BCC consists of two circular conductive loops referred to as end rings, a number of conductive straight elements called rungs or legs, and lumped capacitors on the rungs, end rings or both [24]. According to the position of these capacitors in the coil layout, three types of coils can be created: low-pass, high-pass or band-pass. To generate the desired homogeneous RF field in the BCC when operating at a specific Larmor frequency, currents in the rungs must be sinusoidal to function in Mode 1 [25]. The generation of a sinusoidal current distribution in the rungs and the desired homogeneous B_1_ field at the Larmor frequency, depends on the capacitors used in the rungs and end rings. It is also desirable to have the working mode of operation away from other modes of operation to be able to tune and match the coil [26].

### Simulation environment

Simulations were used to model monopole arrays and BCCs for MRI of the human head at 3 T and 7 T. Our primary target was to achieve a homogeneous magnetic field in the field-of-view of the coil. All simulations were performed using COMSOL Multiphysics 5.0. Hydrogen Larmor frequencies at 3 T and 7 T are 127.74 MHz and 298.2 MHz, respectively. The designs were optimised through repeated simulations and by systematically changing coil parameters.

We designed both MAAs and BCCs using 4, 8, and 12 elements. BCCs incorporated a single capacitor positioned at the midpoint of each rung. The capacitance value of the capacitors, required to achieve resonance and match to 50 Ω, was found through optimisation within the simulation environment. The length of the BCC was 300 mm and its diameter was 240 mm. The conductors used to build each monopole antenna were 550 mm in length and 2 mm in width for 3 T, and 250 mm in length and 2 mm in width for 7 T. The diameter of both arrays was 240 mm.

A cylindrical saline phantom with a height of 170 mm and diameter of 150 mm was simulated with a dielectric constant of 78 and conductivity of 1.96 S/m. Simulations were also performed for a human head phantom, with a relative dielectric constant of 78 and conductivity of 0.55 S/m, as described previously [27]. Using the simulation environment, we studied the effect of (i) Larmor frequency, (ii) planes and slices, (iii) number of excitation ports, (iv) loading and unloading, (v) electric field and specific absorption rate (SAR), (vi) number of coil and antenna elements and (vii) shielding on the magnetic field homogeneity achieved in the field-of-view for each design.

### Monopole antenna array simulation

**Fig 2:**
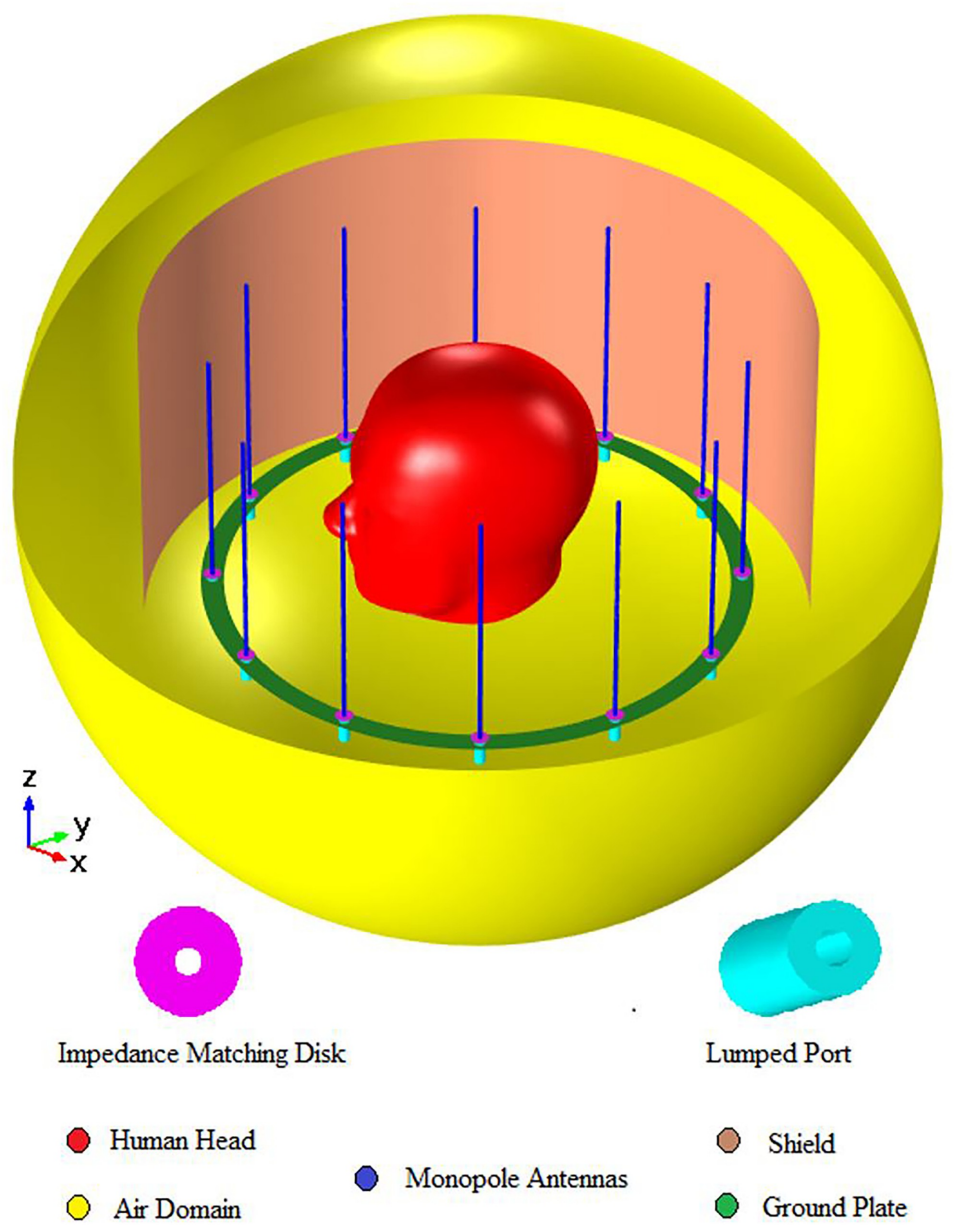
The layout of the monopole antenna array within the spherical simulation domain.

The example MAA shown in Fig 2 has 12 monopole antennae mounted on a dielectric substrate. Separate coaxial lumped ports were used to feed each of the monopole antennae. Excitation was achieved using a single port, two ports (quadrature mode), and all ports (birdcage or circular polarization mode). The exterior conductor of each coaxial port is connected with the ground plate situated on the bottom of the dielectric substrate. The dielectric constant of the substrate was set to 3.38. Our simulation used Teflon, which has a dielectric constant of 2.1, as the material used to fill the space between the inner and outer conductor of the coaxial cable. The angular difference between antenna elements was 30^o^. Increasing the number of elements decreases the angular difference, increasing antenna gain but also producing unwanted grating lobes [28]. For impedance matching, we simulated circular metallic patches etched on the top of the substrate and connected to the corresponding monopole radiator. These patches compensate for the inductance introduced by the monopole arrangement and allow impedance matching to the reference impedance of 50 Ω. All conductive components of the array were modelled as perfect electric conductors. We placed the antenna in a spherical domain and automatically controlled fine meshing was used for domain decomposition.

### Birdcage coil simulation

**Fig 3:**
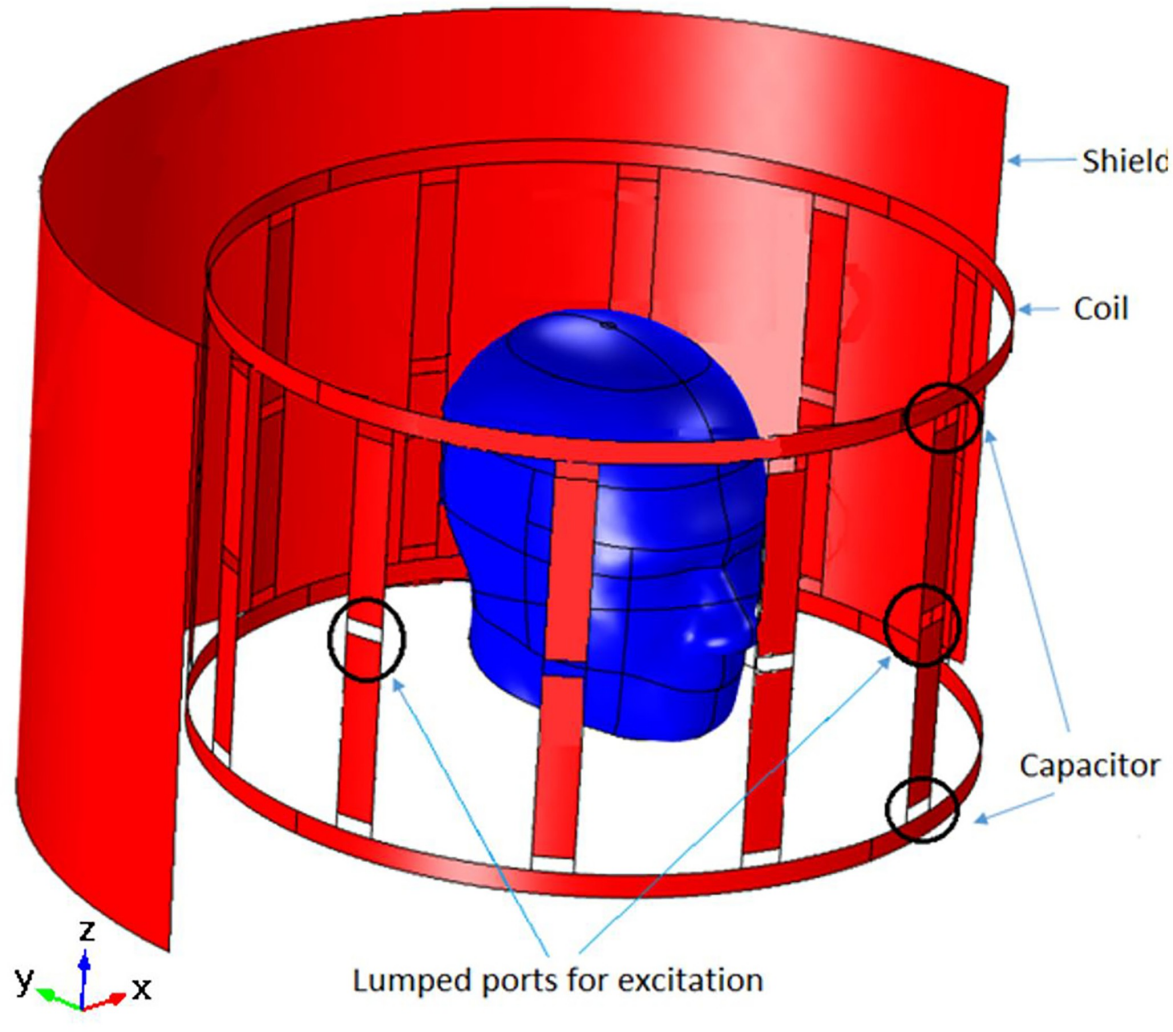
Geometry of the birdcage coil used in the simulations showing the field-of-view of imaging of the head phantom.

Fig 3 shows the geometry of the BCC and how it is positioned around the human head phantom. The capacitors in the rungs were used to change the resonant frequency of the coil and field homogeneity inside the field-of-view. RF shielding was placed outside the coil. Coil surfaces and the shielding were considered to be perfect electric conductors. Lumped ports were used to feed the coil, while the capacitors were demarcated using lumped elements. The absorbing boundary condition was used on the surface of the spherical domain in which the coil was simulated. The capacitors were varied via a parametric sweep. Field homogeneity was assessed by calculating the standard deviation of the magnetic field throughout the phantom. Automatic meshing was used and the maximum mesh element size was limited to 1/6^th^ of free-space wavelength. Simulations using a human brain phantom were also performed.

### Specific absorption rate

With respect to coil design, excessive SAR values can lead to undesirable or dangerous heating of objects within the magnetic field [29]. The SAR value can be calculated as [30]:

$$\mathrm{SAR}=\frac{\sigma {\left|E\right|}^{2}}{2\rho },$$ (Eq 2)

where *σ*, *E* and *ρ* are the tissue conductivity, total electric field and its density, respectively. From Eq 2 it can be seen that SAR is proportional to the square of the electric field. Hence, we produced maps of the electric field as a surrogate for SAR.

### Fabrication

**Fig 4:**
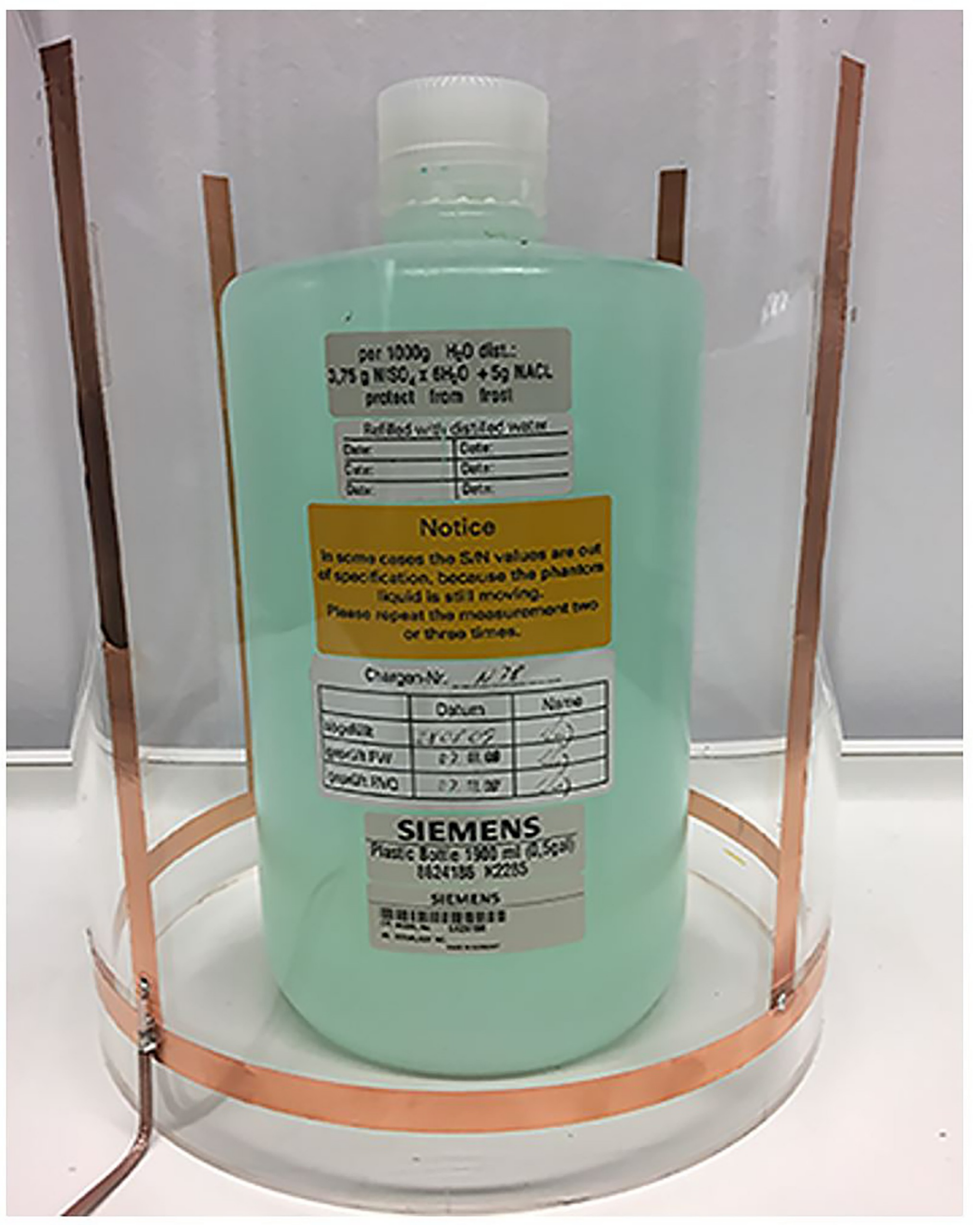
The fabricated 4 monopole antenna array with the Siemens phantom inserted inside the coil.

A monopole array with 4 monopoles was fabricated using copper strips (Fig 4). The diameter of the array was 200 mm and the height of the antenna was 190 mm. Data were collected using an HP 8712B network analyser.

## Results

### 3T and 7T magnetic field homogeneity

**Fig 5:**
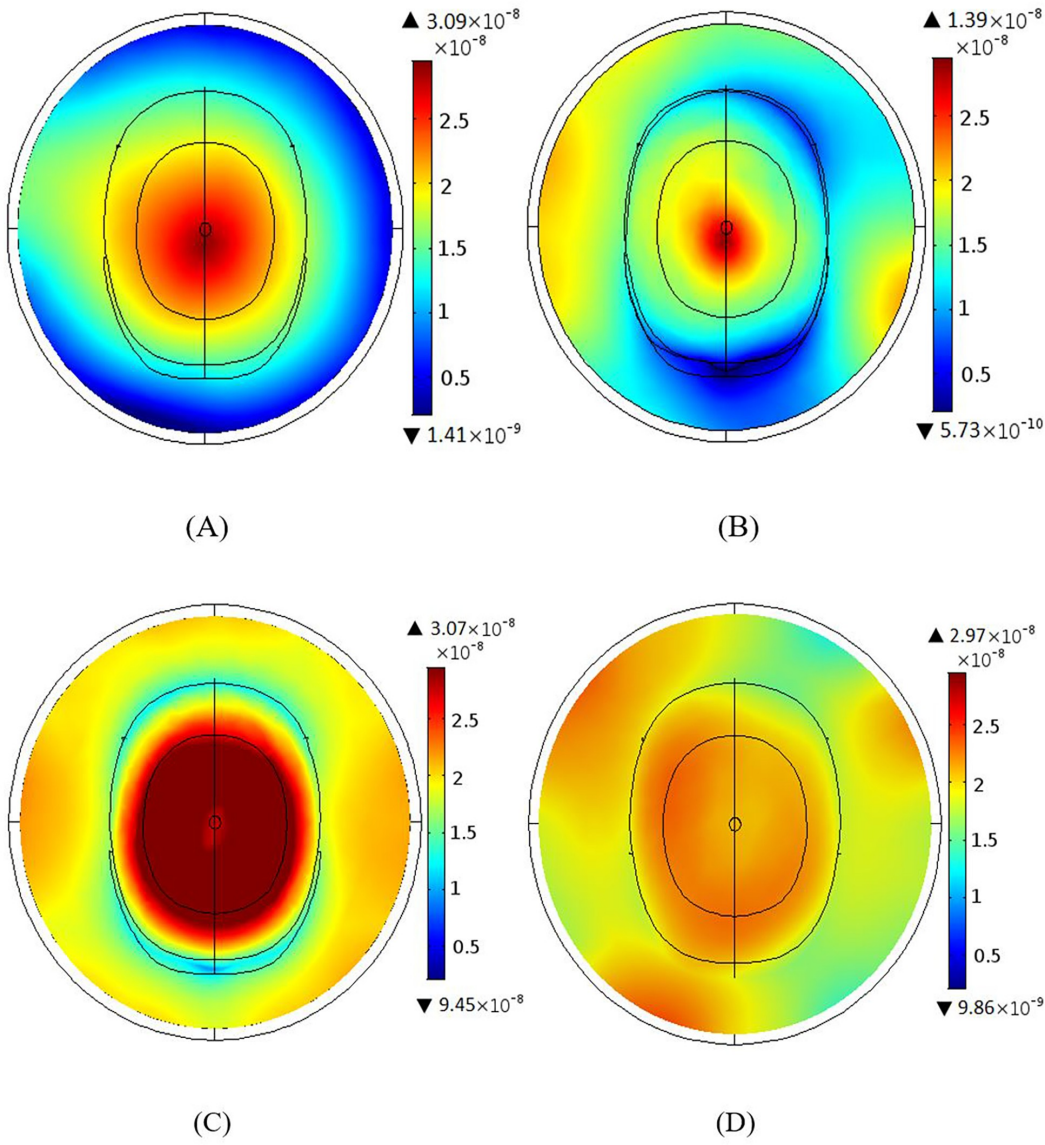
The B_1_ field intensity maps in the presence of the head phantom. (A) The B_1_ field intensity for the BCC design at 3 T. (B) The B_1_ field intensity for the BCC design at 7 T. (C) The B_1_ field intensity for the MAA design for head at 3 T. (D) The B_1_ field intensity for MAA design at 7 T.

Both the BCC and MAA designs were configured using 12 elements with all ports excitation. Fig 5A and Fig 5B show the magnetic fields for the human head phantom for the 3 T and 7 T BCCs. The BCC achieved better homogeneity throughout the centre of the head at 3 T in comparison to 7 T. The field near the coil was better at 7 T as a dark region was visible at 3 T. The 3 T MAA design has better field homogeneity both inside and outside the head and a larger coverage than the 7 T MAA design, as illustrated in Fig 5C and Fig 5D. In comparison to the BCC design (Fig 5A and 5B), the MAA design (Fig 5C and 5D) at 3 T shows better field homogeneity, both inside and outside the head, and a larger region of RF field homogeneity.

### Magnetic field homogeneity across different planes

**Fig 6:**
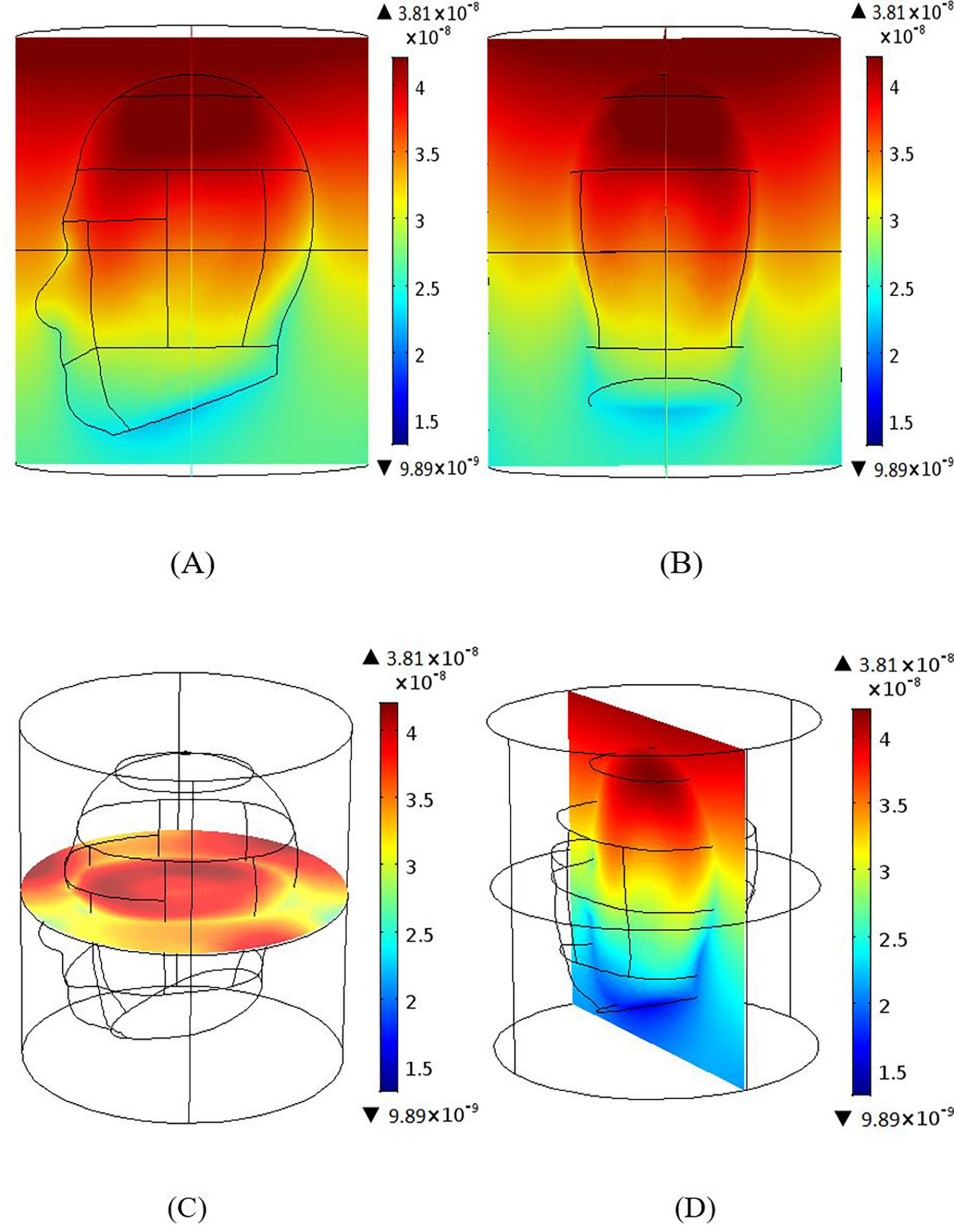
B_1_ magnetic field profiles produced by the MAA design at 7T; sagittal, coronal and axial centre plane view are provided. (A) Sagittal and (B) coronal views of the B_1_ magnetic field distribution. (C) The B_1_ field axial plane. (D) The B_1_ field for the middle slice in the yz-plane.

**Fig 7:**
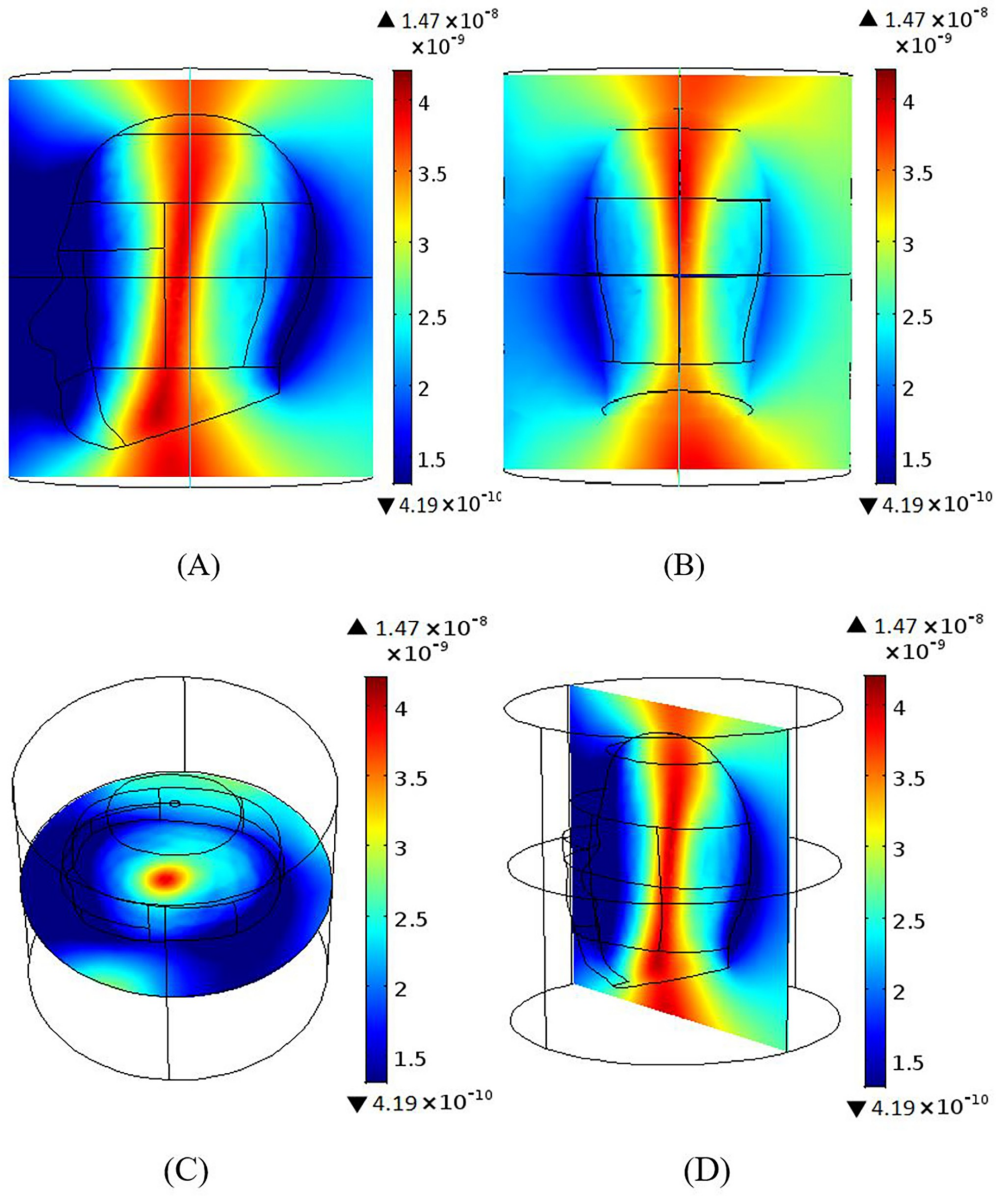
The B_1_ magnetic field distribution generated based on the 7 T BCC design; views sagittal, coronal and axial centre plane views are shown. (A) Sagittal and (B) coronal views of the B_1_ magnetic field. (C) The B_1_ field intensity for the middle slice in the xy-plane. (D) shows higher homogeneity at the centre of the head.

**Fig 8:**
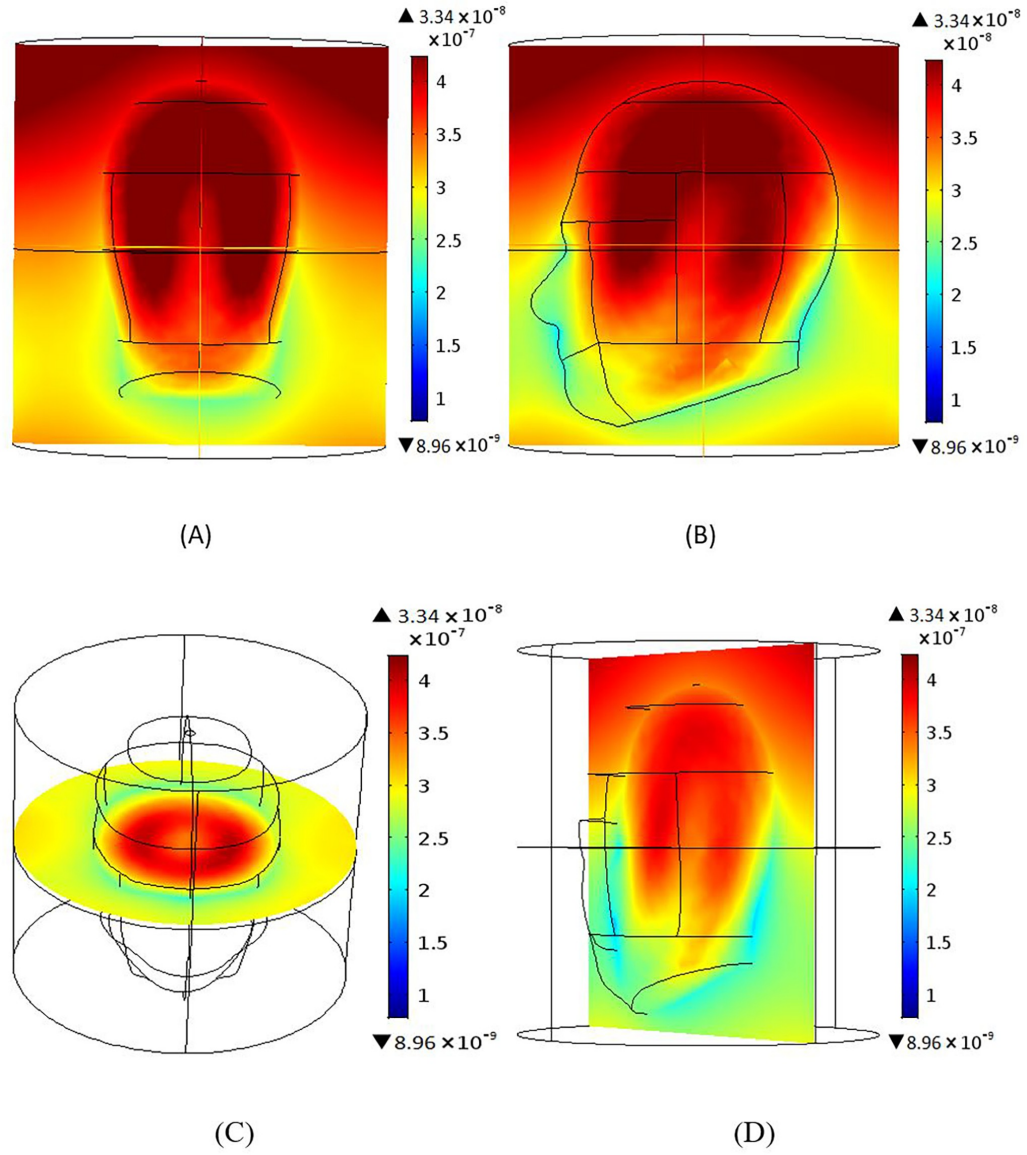
The B_1_ magnetic field intensity maps produced based on the 3 T MAA design; sagittal, coronal and axial centre plane views are shown. (A) Sagittal and (B) coronal views of the B_1_ magnetic field intensity. The B_1_ field for the middle slice in the xy-plane (C) shows high field intensity in the centre of the head in comparison to regions outside of the head. B_1_ field findings for yz-plane (D) are similar.

**Fig 9:**
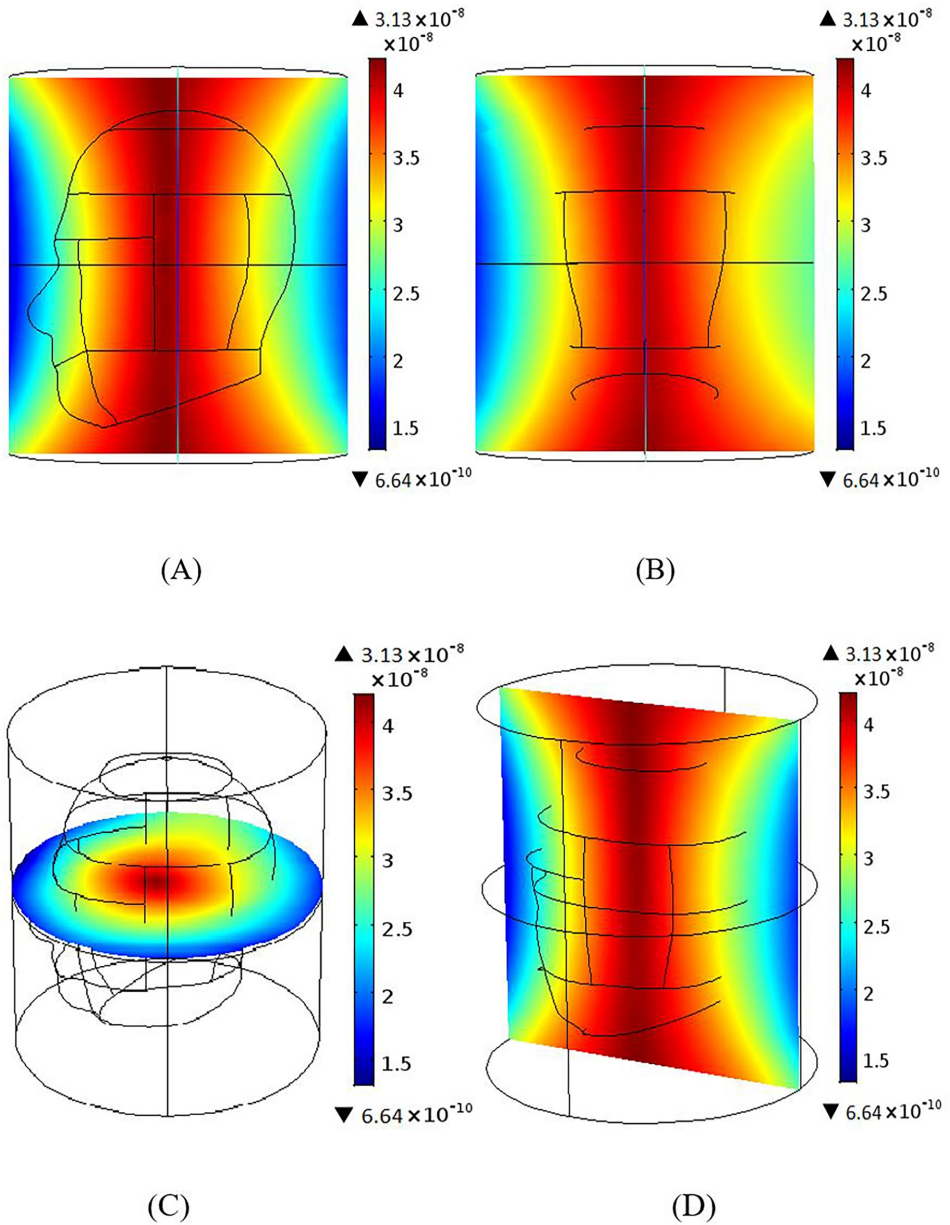
The B_1_ field generated using the 3 T BCC design; sagittal, coronal and axial centre plane views are shown. (A) Sagittal view and (B) coronal views of the B_1_ magnetic field distribution. (C) The B_1_ field for the middle slice in the xy-plane shows higher B_1_ field intensity inside the head than outside the head. The field intensity for the middle slice in the yz-plane (D) is similar.

Results were produced using 12 element BCC and MAA designs with all ports excitation. Figs 6–9 show the B_1_ field homogeneity for sagittal and coronal imaging planes at 3 T and 7 T, using the MAA and BCC designs. Field homogeneity both inside and outside the head phantom for the 7 T MAA is shown in Fig 6A and 6B. The central planes in Fig 6C and 6D indicate that the centre of the head phantom experiences a high B_1_ field. In comparison, the field intensity for sagittal and coronal planes for the 7 T BCC (see Fig 7A and 7B) is less homogeneous. The result for the central plane across the head phantom for the 7 T BCC indicates that the field intensity is lower than the 7 T MAA.

At 7 T, the field intensity produced by the MAA design is almost 2.5 times higher than that produced by the BCC design. At 3 T, the field intensity for the MAA is almost 1.5 times higher than for the 3 T BCC. Homogeneity within the head is greater for 3 T for both MAA and BCC designs than for the 7 T counterparts. In the remainder of the paper we further evaluate the 3 T MAA and BCC design characteristics to be able to expand on the B_1_ field homogeneity results provided to this point with potential application in 3 T clinical MRI instruments.

### Magnetic field homogeneity as a function of coil elements

**Fig 10:**
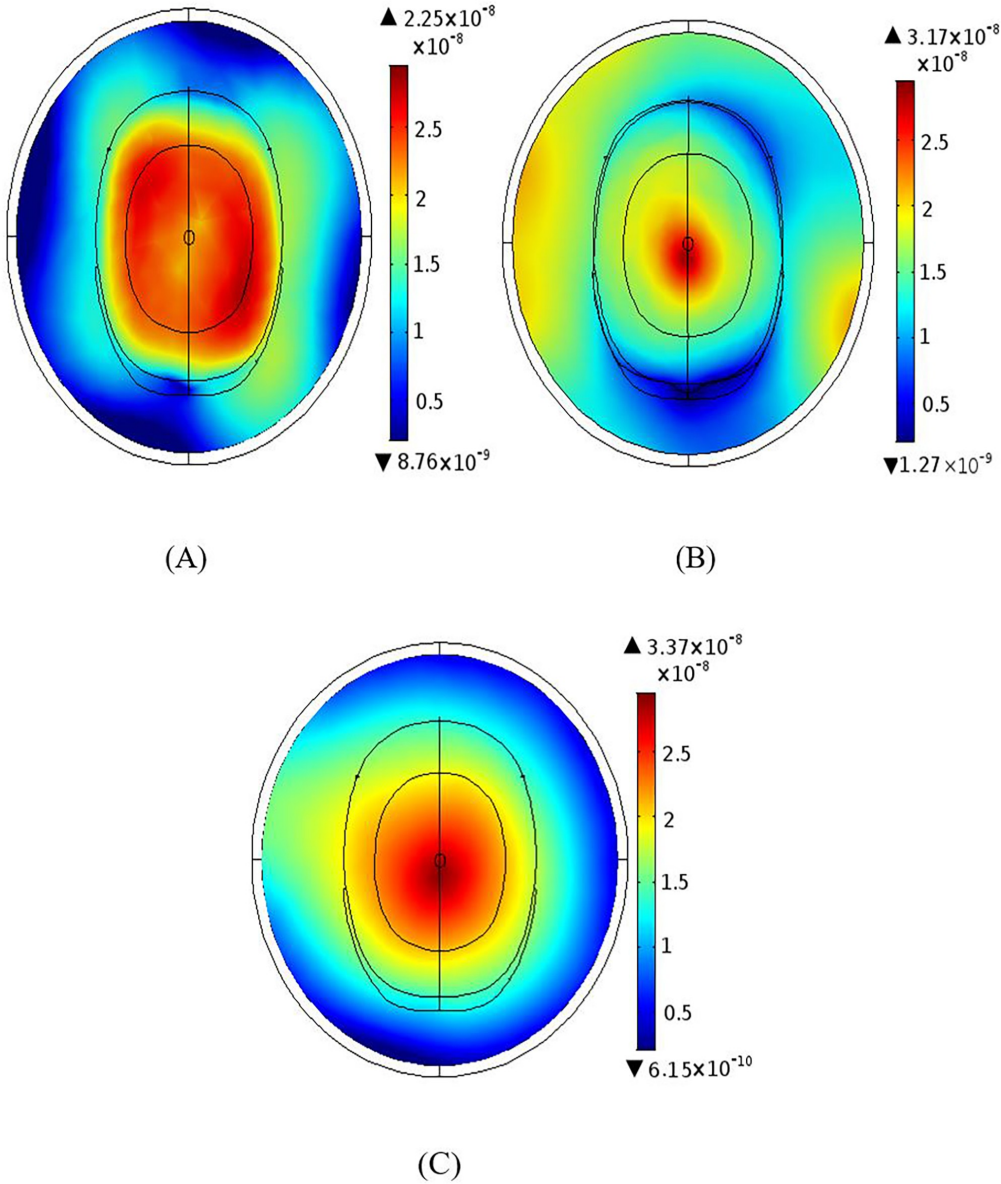
The B1 field homogeneity of the 4, 8 and 12 element 3 T BCC designs. (A) The B_1_ field distribution for the 4 element BCC design with elements separated by 90°. (B) The B_1_ field for the 8 element BCC design with elements separated by 45. (C) The B_1_ field distribution for the 12 element BCC design with elements separated by 30.

**Fig 11:**
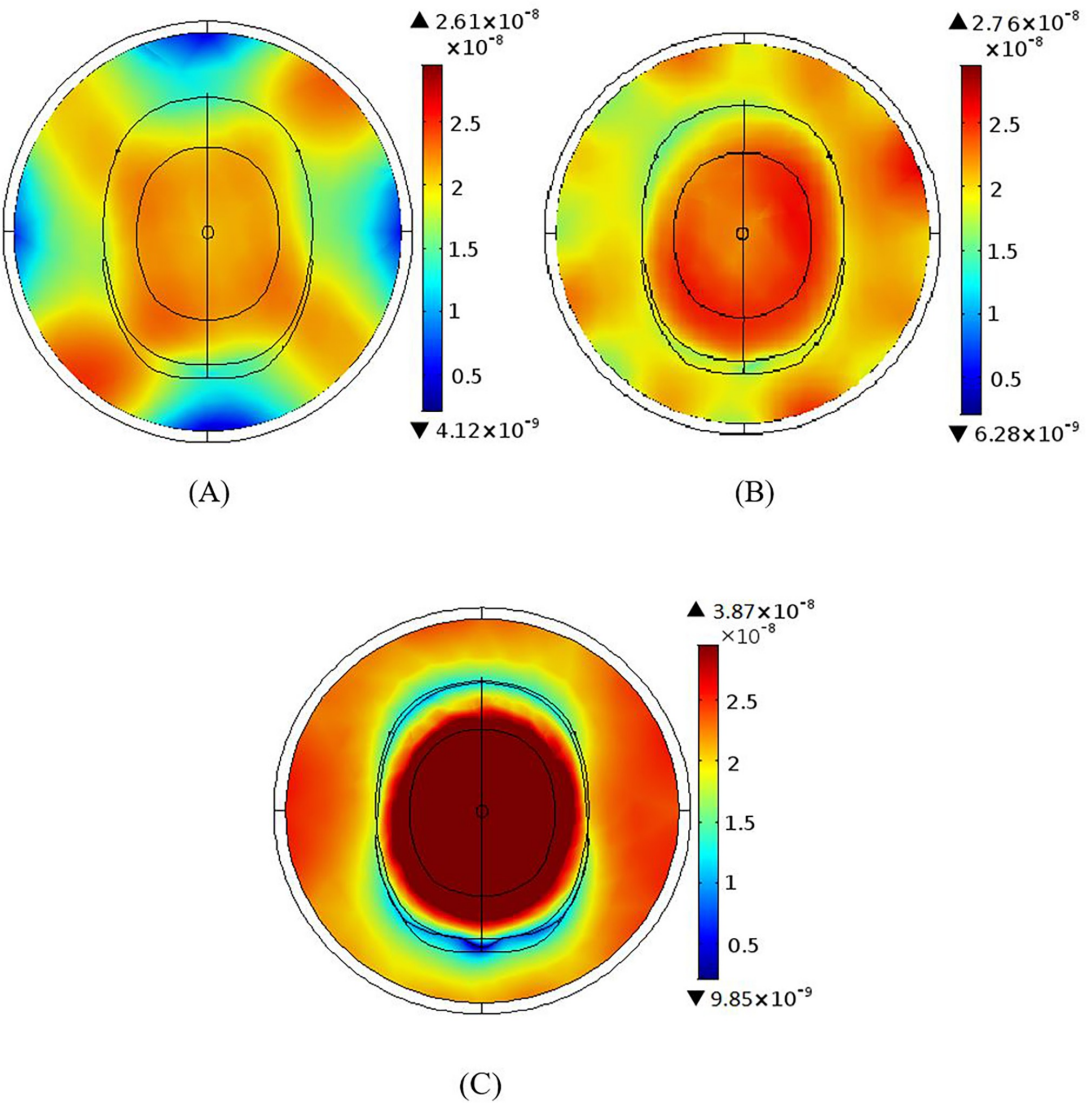
The B_1_ field homogeneity of the 4, 8 and 12 element 3 T MAA designs. (A) The B_1_ field distribution for the 4 element 3T MAA design. (B) The B_1_ field for the 8 element MAA design. (C) The B_1_ field distribution for the 12 element MAA design.

Fig 10 shows the RF homogeneity for the 3 T BCC design with 4, 8 and 12 elements. We showed that the RF field intensity produced by the 8 rung BCC can be 40.9% larger than that generated by the 4 rung coil and, the 12 rung coil produces a 6.3% larger field intensity than the 8 rung coil. For the MAA design (Fig 11), the 8 element array delivers 5.7% larger RF field intensity than the 4 element array, and the 12 element array produces a 40.2% larger RF field intensity than the 8 element array. Irrespective of the design, the 12 element configuration produces the largest RF field intensity and the 12 element MAA produces a 14.8% greater RF field intensity than the 12 element BCC design.

### Effect of excitation ports

Here we examine the effect of changing the number of excitation ports in the MAA and BCC designs at the 3 T MRI frequency. Both the MAA and BCC designs were driven in three different modes. First is single element excitation where the whole coil was excited using only one port. Second is the quadrature excitation using two ports of same magnitude with a 90° phase difference between them. The third one is all port excitation. Here, the magnitude is same for the excitation but have a phase delay of 360°/*N*, where *N* is the number of elements in the coil. As an example, for 12 elements coil the phase difference will be (0, 30, 60, …. 330).

**Fig 12:**
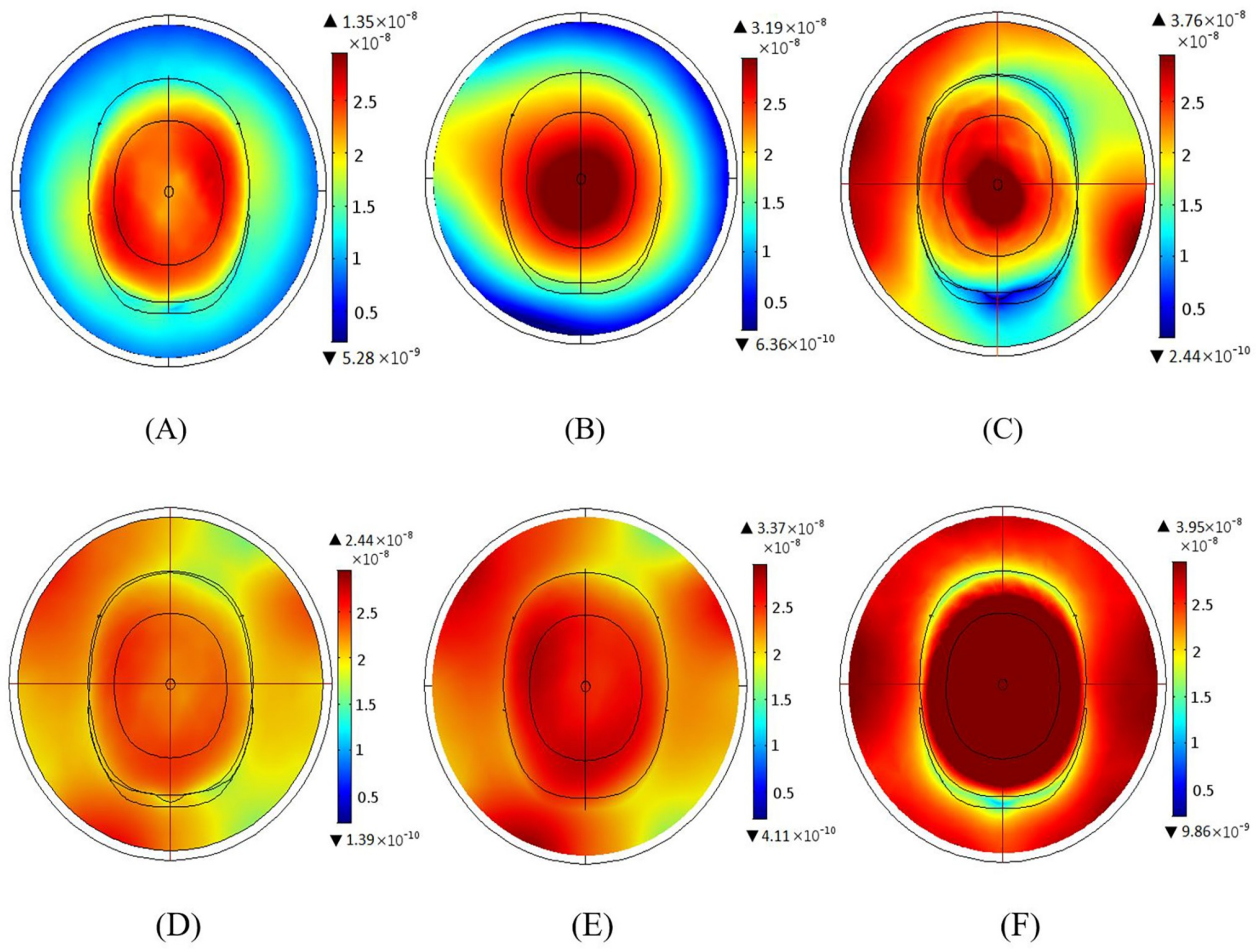
The B_1_ field distributions for single, quadrature and all port excitations for the BCC and MAA designs. (A) B_1_ field distribution for single port excitation of the BCC design; a homogeneous field is produced across the head. (B) B_1_ field for quadrature BCC excitation; the maximum intensity achieved using the quadrature BCC excitation is almost 2.5 times higher than the single port excitation. (C) B_1_ field for all port BCC excitation; the field is fairly uniform both inside and outside the head. (D) B_1_ field distribution for single port MAA excitation. (E) B_1_ field distribution for quadrature MAA excitation. (F) B_1_ field for all port MAA excitation. When compared to the single and quadrature port MAA excitation simulations, the MAA field achieved using all port excitation is homogeneous throughout the head.

Fig 12 illustrates the RF field distributions for single, quadrature and all port excitation for the BCC (Fig 12A–12C) and the MAA (Fig 12D–12F) designs. Field intensity increased with a greater number of excitation ports for both BCC and MAA designs. For the BCC design, the maximum value of the RF field intensity using quadrature excitation (Fig 12B) was 136.0% larger than for single port excitation (Fig 12A), and RF field intensity for all rung excitation (Fig 12C) was 17.9% larger than for quadrature excitation. For the MAA design, quadrature excitation (Fig 12E) resulted in 38.1% larger field intensity than single port excitation (Fig 12D), and all element excitation (Fig 12F) led to 17.2% larger field intensity than quadrature excitation. The RF field intensity produced by the MAA design was 80.7%, 5.6%, and 5.1% higher than that produced by the BCC design for single, quadrature, and all rung excitation, respectively.

### Effect of shielding

**Fig 13:**
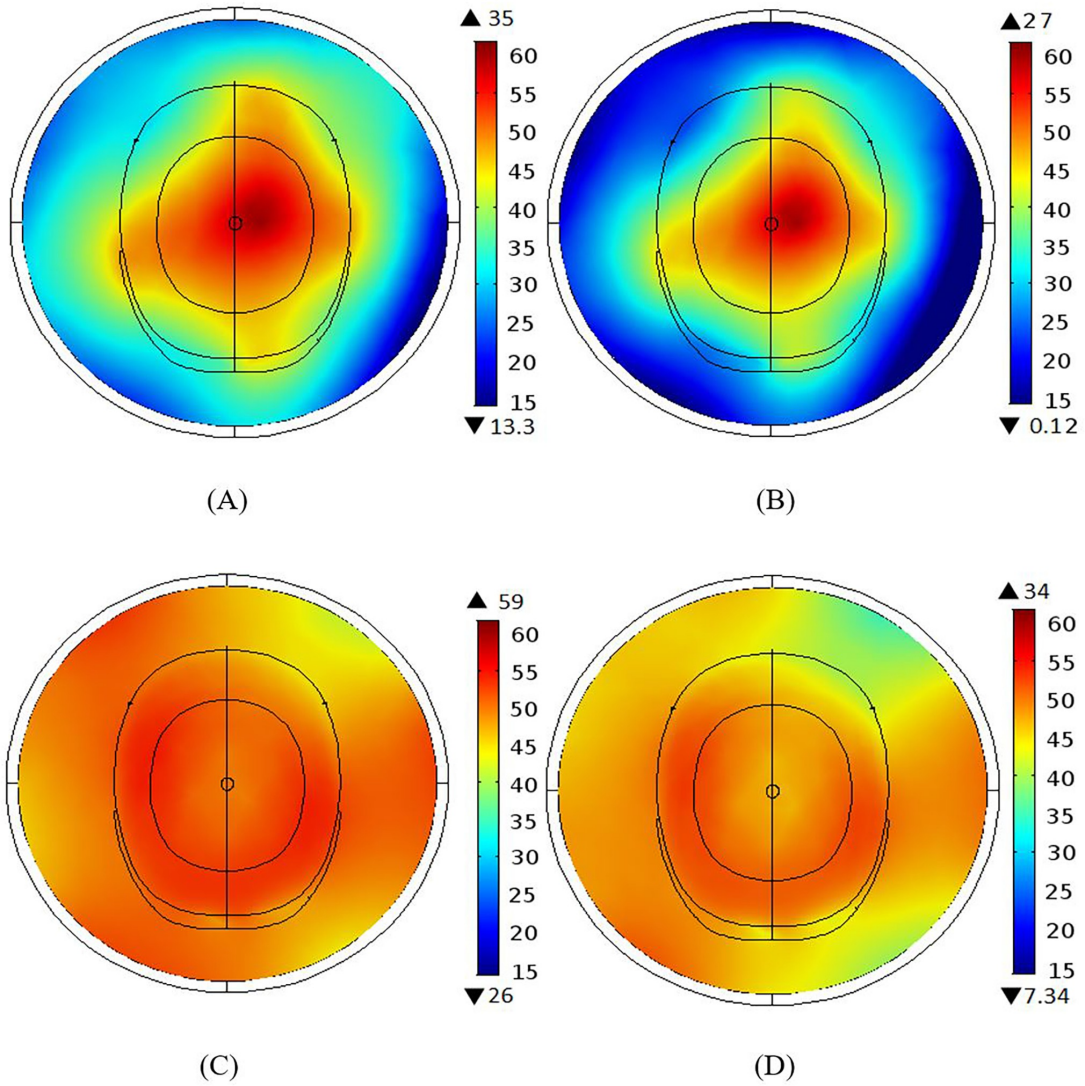
The effect of shielding the 3 T BCC and MAA designs. (A) Magnitude of the B_1_ field for the shielded BCC design. (B) Magnitude of B_1_ field for the unshielded BCC design. (C) Magnitude of B_1_ field for the shielded MAA design. (D) Magnitude of B_1_ field distribution for the unshielded MAA design. Shielding in general increases the magnitude of the field.

Fig 13 illustrates the effect of shielding the BCC and MAA designs at the 3 T MRI frequency. Fig 13A and 13B show the magnetic field intensity of the BCC design for the shielded and unshielded simulations. Similarly, Fig 13C and 13D show the results for the shielded and unshielded MAA design simulations.

Quality within the field-of-view, as measured by field distribution, improved with the use of shielding. For the BCC design, shielding led to a 29.6% higher field intensity than the unshielded design, and field homogeneity also increased. For the MAA design, the use of shielding increased field intensity by 73.5%. Overall, we found that the MAA design produces almost twice the magnetic field intensity of the BCC design, irrespective of whether coils are shielded or unshielded.

Table 1 summarises the characteristics of the magnetic field intensity within three different sized field-of-views (FOVs). Whilst for the BCC design shielding does not lead to a better field homogeneity as measured using the standard deviation, the MAA design benefits greatly from the use of shielding. In fact, the standard deviation decreases by more than a factor of two with the use of shielding, and it is also about a factor of two better than in the BCC design. The table also shows that as the FOV size decreases, the mean magnetic field intensity increases. This suggests that fields decrease away from the centre for the coil. The amount of decrease in mean magnetic field intensity going from a 30mm diameter FOV to a 150mm diameter FOV is 23% and 13% for the unshielded and shielded MAA design, and 40% and 23% for the unshielded and shielded BCC design. In addition, the use of shielding increase the mean magnetic field intensity by an amount less than 100% in the case of the BCC design, and more than 120% for the MAA design (i.e. FOV_15_ result). These results suggest that the shielded MAA design achieves the largest field sensitivity.

**Table 1:** Magnetic field intensity statistics for the unshielded and shielded BCC and MAA designs. The diameter of the FOVs are FOV_3_ = 30mm, FOV_7_ = 70mm and FOV_15_ = 150mm all of which are centred on the same position.

	Array	FOV	Magnetic field intensity (x10^-8^ T)			
			max	min	mean	std
**Unshielded**	BCC	FOV_3_	1.770	0.615	1.192	0.577
		FOV_7_	1.540	0.355	0.947	0.592
		FOV_15_	1.320	0.118	0.719	0.601
	MAA	FOV_3_	2.035	0.871	1.453	0.582
		FOV_7_	1.970	0.715	1.342	0.627
		FOV_15_	1.780	0.470	1.125	0.655
**Shielded**	BCC	FOV_3_	2.250	1.230	1.740	0.510
		FOV_7_	2.140	1.015	1.577	0.562
		FOV_15_	1.923	0.770	1.346	0.576
	MAA	FOV_3_	3.150	2.640	2.895	0.255
		FOV_7_	2.930	2.410	2.670	0.260
		FOV_15_	2.790	2.250	2.520	0.270

### Magnetic field for different loading

**Fig 14:**
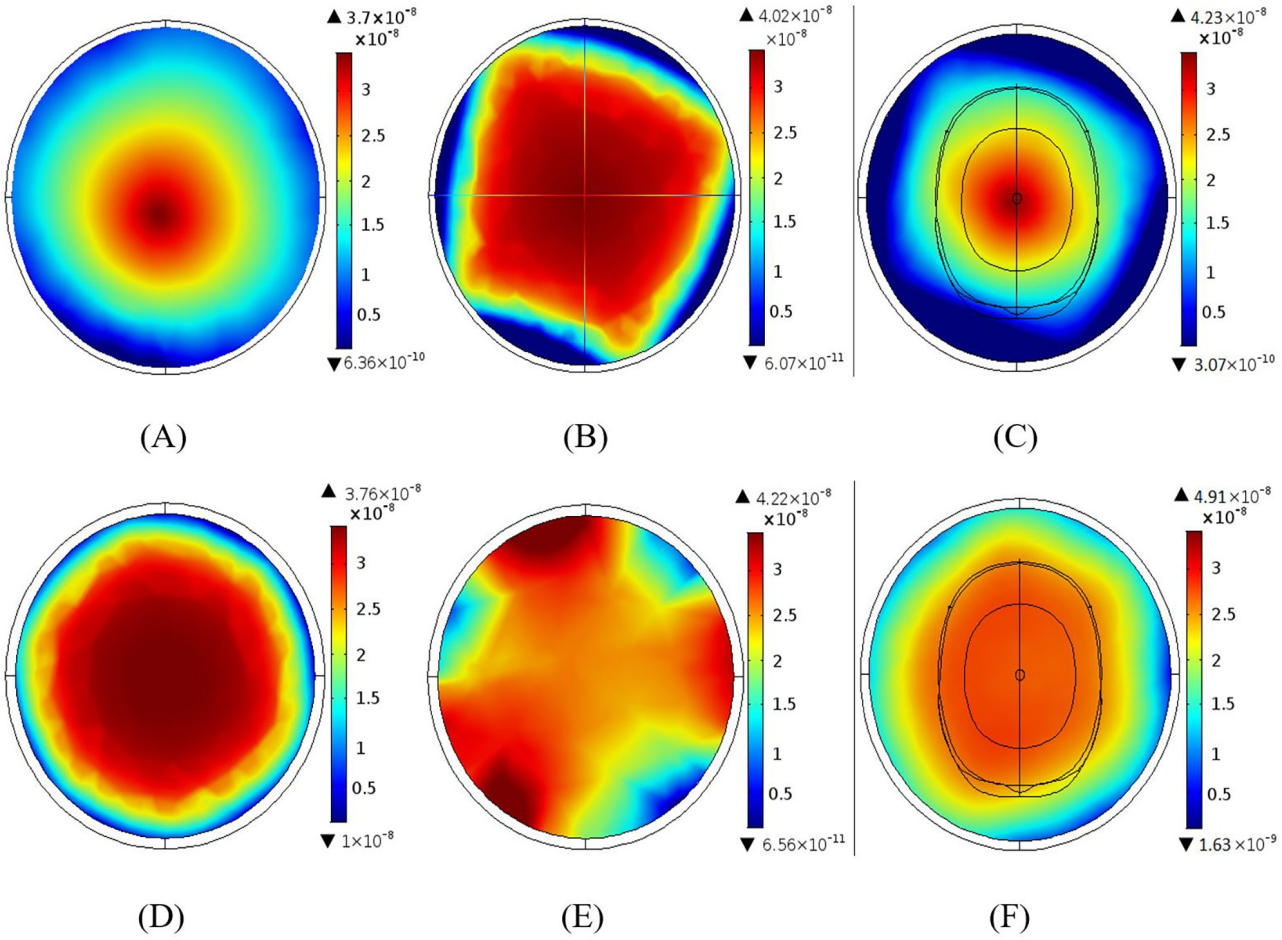
The B_1_ field distribution at 3 T using BCC and MAA designs when the coil was unloaded, loaded with saline phantom and loaded with the human head phantom. (A) The B_1_ field distribution for the unloaded BCC design. (B) The B_1_ field distribution for the BCC design loaded with a saline phantom. Saline has a higher dielectric constant than water, hence homogeneity decreases; expected as the wavelength shortens. (C) The B_1_ field distribution for BCC design loaded with human head phantom. (D) The B_1_ field distribution for the unloaded MAA design. (E) The B_1_ field distribution for MAA design loaded with saline phantom. (F) The B_1_ field distribution for the MAA design loaded with the human head phantom; the field is relatively homogeneous across the phantom with high in the centre.

We tested the 3 T BCC and MAA designs when the coil was unloaded, loaded with saline phantom and loaded with the human head phantom. Fig 14A–14C shows the RF field distribution for the unloaded BCC design (Fig 14A), the saline phantom (Fig 14B) and for the human head phantom (Fig 14C). Fig 14D–14F shows the field distributions for the MAA design for each loading condition. Our simulated MAA design outperformed the BCC design when a head phantom was placed in the coil FOV.

### Analysis of specific absorption rate

**Fig 15:**
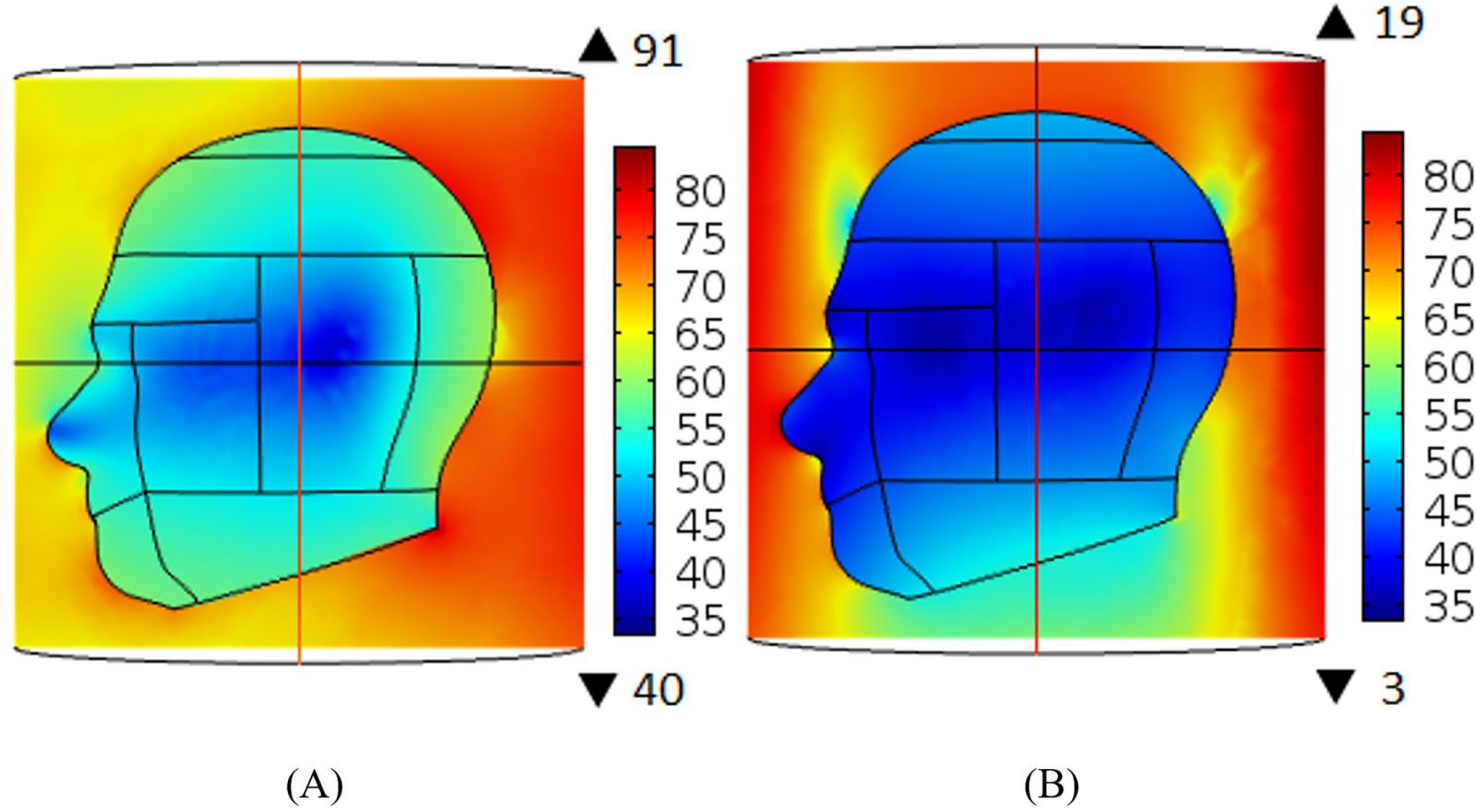
The electric field distribution inside the head phantom. Shown are the results for the BCC (A) and MAA (B) designs. A marked decrease in power deposition can be achieved using the MAA design.

Simulations were performed at 3 T MRI frequency using 8 elements for both the MAA and BCC designs. The MAA and BCC designs were not shielded. Fig 15 illustrates the electric field distribution for the MAA (Fig 15A) and BCC (Fig 15B) designs. It is shown in the figures that the BCC design produced the highest electric field of 84.5 V/m, while the MAA design had a maximum electric field of only 14 V/m. According to Eq 2, a higher electric field leads to a larger SAR value.

### Experimental results

#### Matching analysis

**Fig 16:**
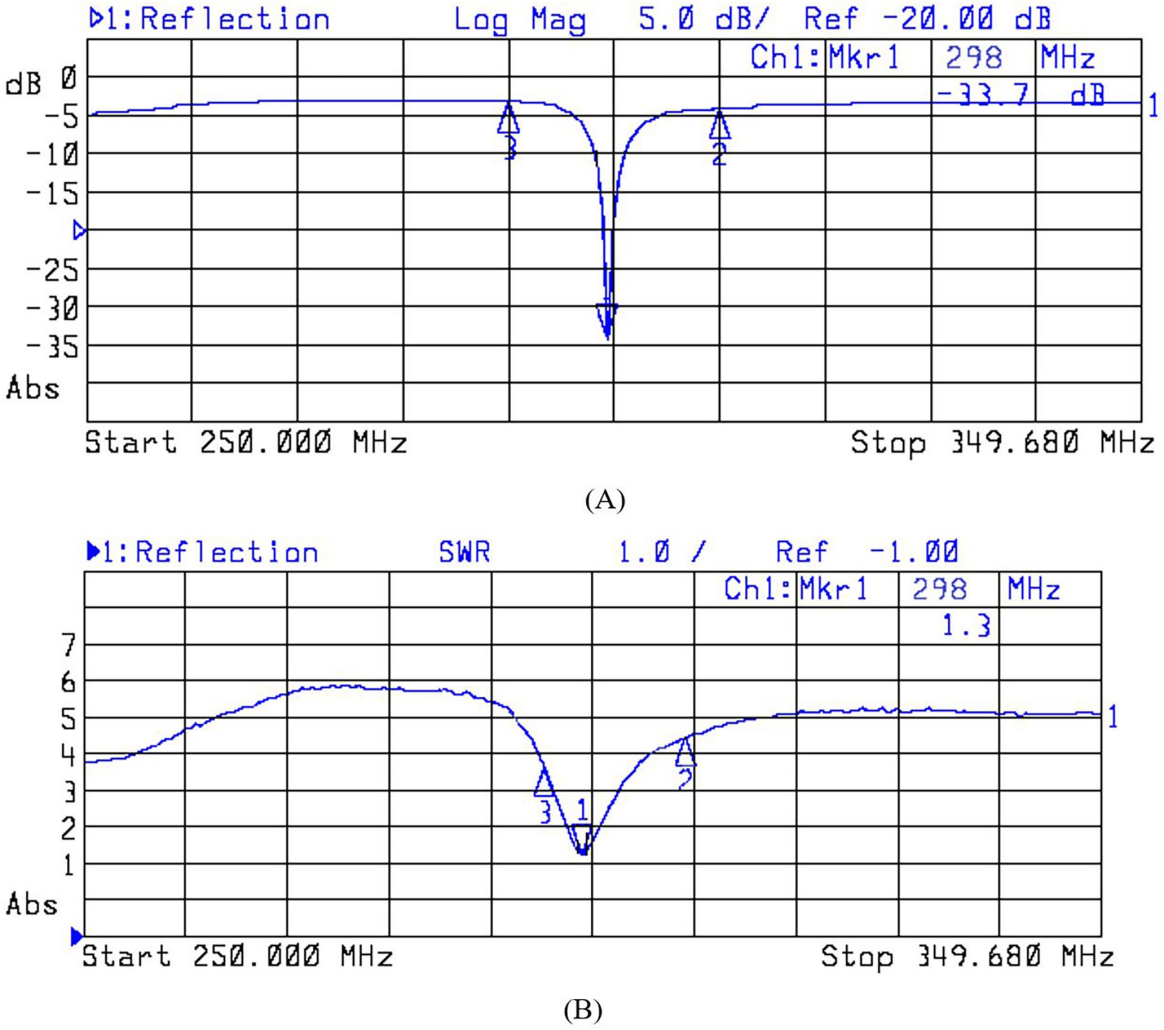
The experimentally measured S_11_ values and the standing wave ratio (SWR) for the fabricated 7 T MAA design. (A) S_11_ and (B) SWR measured at 298 MHz, the 7 T frequency.

Lumped port excitation was used and therefore it was important to assess the matching and tuning of the array. This section describes the S parameter value (S_xy,_ which denotes the interaction between the x^th^ and y^th^ antenna elements) which is used to evaluate matching and tuning performance. S_11_ represents the return loss of the antenna, indicating minimal return loss in the fabricated MAA. Fig 16 shows the values for the S_11_ and the standing wave ratio (SWR). The unloaded antenna array has an S_11_ value of -33.7 dB and a SWR of 1.3 at a frequency of 298 MHz.

#### Coupling analysis

**Fig 17:**
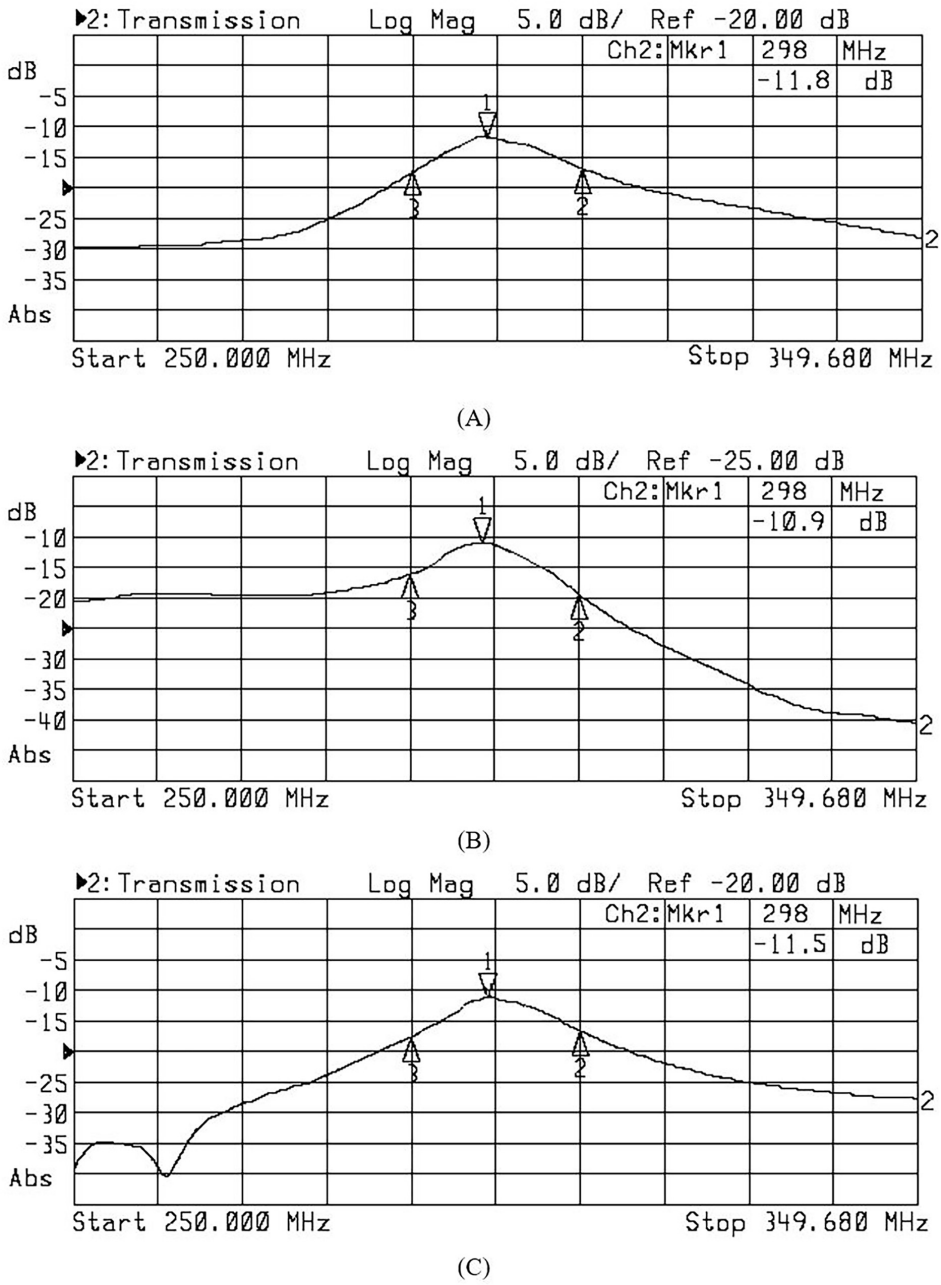
The experimentally measured S-parameter values for the fabricated 4 element monopole antenna array (MAA) at 7 T. (A) S_21_, (B) S_31_, and (C) S_41_.

Mutual coupling results are shown in Fig 17. Within the circular array, the angular distance between elements 1, 2, 3 and 4 is 90°, 180°, and 270°, respectively. The maximum value of S_21_ is -11.8 dB, of S_31_ is -10.9 dB, and of S_41_ is -11.5 dB.

## Discussion

We investigated the potential use of MAAs with the aim of improving radio frequency coil field homogeneity and reducing power deposition. We investigated how the number of array elements, shielding and loading influence the field homogeneity and specific absorption rate and, compared MAA and BCC simulation results of different number of rungs at both 3 T and 7 T. In addition, we fabricated a 4 element 7 T MAA and performed bench testing to assess coil sensitivity and decoupling between elements. As expected, field homogeneity degrades with an increase in field strength, however by increasing the number of elements and number of excitation ports, some loss in field homogeneity can be recovered. With the use of shielding, field homogeneity can be improved further. We found our MAA design to have a higher sensitivity than the BCC design, implying lower transmit power can be used and thereby the specific absorption rate reduces as well. Via bench testing we were able to show good overall sensitivity and decoupling between elements.

### Simulation findings

Degradation of field homogeneity with increasing the field strength from 3 T to 7 T is not unexpected since wavelength is shorter at higher fields [31]. Our results suggest that field homogeneity can be improved through the use of greater number of MAA elements (Fig 10 and Fig 11). As the magnetic field produced by the antenna array is the superposition of fields created by individual elements, an increase in the number of array elements results in smaller separation between elements, in turn leading to improved field homogeneity. Whilst this is true, a smaller distance between elements also leads to increased coupling between elements, which reduces overall coil efficiency. For this reason a compromise between how many elements are used in the design and what level of mutual coupling is acceptable has to be reached. This trade-off between field homogeneity and mutual coupling can somewhat be overcome by using a smaller number of elements and increasing the number of excitation ports (Fig 12), as fields in individual elements become more stable through the use of more excitation ports. The best field homogeneity is achieved when all elements are excited individually. We should point out that in the MAA design with all element excitation, the phases between excited elements may be different, which can degrade field quality. Therefore, excitations of elements should be phased correctly to ensure that the best quality field can be delivered to the load. This might also be an advantage, as radio frequency field shimming could be used with the MAA (i.e. by adjusting excitation field amplitude and phase for each element [32]), whilst in the BCC design this is not possible as rungs are connected via an end ring and the amplitude and phase of the field produced by each rung cannot be changed by changes in the input signal amplitude and phase.

The addition of shielding to the MAA can lead to two desirable outcomes (Fig 13). Firstly, it can improve the field strength of the coil, which can be interpreted as lower power requirements for transmission and higher sensitivity for signal detection. Secondly, the use of shielding allows shaping of the field and we were able to demonstrate increased field coverage in the axial coordinate direction, as was shown previously [13].

The specific absorption rate of radio frequency energy in tissue increases with increased electric field strength either as a consequence of the coil design or increased input power, duty cycle and transmitter coil type, whilst increased loading generally leads to lower power deposition per unit volume. Additionally, field inhomogeneity can lead to the creation of localised hot spots, where high amounts of electric field concentrate to a specific region [33]. It is therefore important to design radio frequency coils with the highest level of field homogeneity. In comparison to the BCC design, the MAA design was able to deliver a higher level of field homogeneity across the imaging volume, thereby reducing the likelihood of localised tissue heating effects. We attribute this gain in field homogeneity to differences in how different designs are excited. That is, the BCC design used quadrature excitation and the MAA design used all port excitation. Quadrature excitation is applied on one end ring of the BCC configuration and not the other, hence larger fields are produced near the ports and lower on the other end ring. This effect becomes more prominent as coil frequency increases.

### Experimental findings

At MRI frequencies tuning is mostly performed through the use of variable capacitors, and geometry variations are used to a lesser extent [34]. Matching is usually controlled using a lumped-element circuit, although transmission-line schemes could be used instead [34]. We varied the geometry of the configuration for the purpose of tuning and matching. We were able to reach a good level of tuning and matching through successive adjustments of element lengths (tuning) and position of the feed point along the ground plate (matching). All elements were kept the same length. The standing wave ratio (SWR) has been routinely used to measure how much input power is reflected. For systems fed using a 50 Ω coaxial cable and via the ground plane, a good level of matching can be achieved when SWR < 1.5 and return loss, S11, < -14dB [35]. Our fabricated 7 T MAA was able to deliver a performance better than the benchmark (Fig 16).

The effectiveness of an antenna array relies on the level of independence achieved between individual antenna elements, and in reality, mutual coupling exists between elements. Mutual coupling should be minimised if it cannot be removed totally. A level of -10dB coupling between any two elements has been shown to be adequate for MRI applications [36]. A various number of decoupling methods have been developed, including the circuit voltage and receiving mutual impedance methods, lumped elements networks and magnetic wall decoupling, and these have been used in radio frequency application and antenna design [37,38]. We opted not to incorporate decoupling for individual elements, since the 4 element MAA design was able to achieve a good level of decoupling simply through the spacing of elements and shielding provided by the load (decoupling between one element with respect to other elements ranged between -11.8 dB to -10.9 dB). However, a decoupling method, such as magnetic wall or shielding of individual elements, may be needed when number of array elements is increased.

### Comparison to existing models

Two different MAAs have been proposed for ultra-high field MRI applications. Hong *et al* described an 8 element MAA for 7 T MRI applications [19]. In their design a non-hollow ground plate was used, to which each of the 8 elements was connected. Hence, one side of the array is closed (i.e. at the top of the head) and access to the field-of-view of imaging is through the opposite end. A large ground plate introduces eddy currents, and therefore, in their design the ground plate had to be broken into sections and connected using an excessive number of capacitors. Their mutual coupling values between elements were between -9.3 dB to -10.6 dB, which is higher than for our design. This difference may be explained by the use of 4 additional elements in their MAA, the impact of which is that elements are closer together. Nonetheless, we were still able to achieve reasonable decoupling between elements located at opposite sides of the array (-11.8 dB versus -10.6 dB). They also showed that a MAA can achieve higher and more symmetric sensitivity around the middle of the brain than a surface coil design and, the MAA outperformed a dipole array in terms of sensitivity. The field produced by their layout is stronger at the top of the head, and weaker towards the neck. This is because each element is connected through the ground plate, which has to be at the top of head, and energy dissipates along the element. The field achieved around the middle of the head was significantly higher than around the cortex and skull. In our case, since the ground plane is a circular loop, we can place it near the neck instead of at the top of the head. As such, we were able to produce a more homogeneous field across the brain and we do not have as pronounced field gradient in the axial direction.

Woo *et al* proposed a different 8 element 7T MRI MAA wherein they extended the effective sensitivity in the axial direction through the use of capacitors in each of the elements [18]. They used a ground plate similar to the one used by Hong *et al*, and in addition, shielded each of the elements of the MAA. These modifications did improve the field and sensitivity towards the neck, however mutual coupling between elements increased (in the range -7.7 dB to -6.7 dB) above the -10 dB needed to maintain image quality. Our MAA work demonstrates that ground plane placement and how elements are connected play an important role in being able to deliver a high quality transmit field whilst having high sensitivity to the signal.

## Conclusion

A new MAA design has been described and evaluated through simulations and by fabricating a 4 element array for bench testing. Simulation results were compared to the traditional BCC design. The results of bench testing the fabricated MAA were used to analyse tuning, matching and decoupling between individual elements. Simulations were performed at 3 T and 7 T MRI frequencies using 4, 8, and 12 element MAA configurations, and compared to 4, 8 and 12 element BCCs designs. The MAA design is capable of producing a more homogeneous RF field distribution, a comparatively larger magnetic field intensity and a lower electric field (i.e. lower SAR) than the BCC configurations studied and therefore provides promise for head imaging applications. Using the MAA, signals from deep brain structures could be enhanced at ultra-high field. Additional studies are required to investigate how to extend the field-of-view of imaging in the axial direction. This would make such a design even more attractive for brain studies in general.
